# Guidelines for the management of paediatric cholera infection: a systematic review of the evidence

**DOI:** 10.1080/20469047.2017.1409452

**Published:** 2018-05-23

**Authors:** Phoebe C. M. Williams, James A. Berkley

**Affiliations:** a Nuffield Department of Clinical Medicine, The University of Oxford, Oxford, UK; b Kenya Medical Research Institute (KEMRI)/Wellcome Trust Research Programme, Kilifi, Kenya; c The Childhood Acute Illness and Nutrition Network (CHAIN), Kilifi, Kenya; d Centre for Tropical Medicine and Global Health, Nuffield Department of Medicine, The University of Oxford, Oxford, UK

**Keywords:** Cholera, diarrhoea, antibiotics, antimicrobial resistance, paediatric international health, child health

## Abstract

**Background:**

*Vibrio cholerae* is a highly motile Gram-negative bacterium which is responsible for 3 million cases of diarrhoeal illness and up to 100,000 deaths per year, with an increasing burden documented over the past decade. Current WHO guidelines for the treatment of paediatric cholera infection (tetracycline 12.5 mg/kg four times daily for 3 days) are based on data which are over a decade old. In an era of increasing antimicrobial resistance, updated review of the appropriate empirical therapy for cholera infection in children (taking account of susceptibility patterns, cost and the risk of adverse events) is necessary.

**Methods:**

A systematic review of the current published literature on the treatment of cholera infection in accordance with the Preferred Reporting Items for Systematic Reviews and Meta-Analyses (PRISMA) was undertaken. International clinical guidelines and studies pertaining to adverse effects associated with treatments available for cholera infection were also reviewed.

**Results:**

The initial search produced 256 results, of which eight studies met the inclusion criteria. Quality assessment of the studies was performed as per the Grading of Recommendations Assessment, Development and Evaluation guidelines.

**Conclusions:**

In view of the changing non-susceptibility rates worldwide, empirical therapy for cholera infection in paediatric patients should be changed to single-dose azithromycin (20 mg/kg), a safe and effective medication with ease of administration. Erythromycin (12.5 mg/kg four times daily for 3 days) exhibits similar bacteriological and clinical success and should be listed as a second-line therapy. Fluid resuscitation remains the cornerstone of management of paediatric cholera infection, and prevention of infection by promoting access to clean water and sanitation is paramount.

## Introduction

*Vibrio cholerae* is a highly motile, halophilic Gram-negative, comma-shaped bacterium. The main reservoirs of *V. cholerae* are people and aquatic sources such as brackish water and estuaries [1]. *V. cholerae* is serologically classified on the basis of variations in the 0-antigen lipopolysaccharide structure, and, while over 200 serogroups have been identified, only two (*V. cholerae* 01 and 0139) cause cholera epidemics [2].

*V. cholerae* 01 predominates as the cause of cholera globally. This species is further divided into two main serotypes — Inaba and Ogawa serogroups — and two biotypes (El Tor and classical) on the basis of biochemical differences and susceptibility to specific bacteriophages, with the latter now appearing to be extinct [3]. Most environmental *V. cholerae* are not toxigenic. However, the pathogenic strains of *V. cholerae* 01 and 0139 may harbour genes within a filamentous bacteriophage, known as CTXφ that encode for ‘cholera toxin’ (CT) which acts by entering the surface of epithelial cells and increasing cyclic adenosine monophosphate activity, leading to chloride secretion at the apical surface. This results in significant water and sodium losses, leading to the massive fluid and electrolyte efflux that is the hallmark of clinical cholera infection [4].

Cholera is endemic in approximately 50 countries — placing 1.4 billion people at risk — and the vast majority of the clinical burden is borne in resource-limited settings owing to restricted access to clean water sources. Each year, cholera is estimated to cause 3 million cases of diarrhoeal illness worldwide, and up to 100,000 deaths [5]. During epidemics, the case fatality rate is 1–4%, higher in rural areas [2]. Importantly, the burden of cholera has been increasing in the past decade [6]. Patterns of transmission and infection differ between endemic areas (where seasonal distribution occurs after rainy seasons, and the incidence is highest in young children owing to a lack of protective immunity) in contrast with regions which experience cholera epidemics (where attack rates are similar in adults and children) [7]. Superimposed epidemics may also occur in endemic regions in response to fluctuations in population-based immunity and climate [8]. Since the early 1800s, there have been seven cholera pandemics, with the current pandemic (of *V. cholerae* 01 El Tor) commencing in 1961 and continuing in three successive waves — from South Asia to other regions of Asia, the Oceania and Africa [9].

The infectious dose of *V. cholerae* required to cause infection is relatively high (over 108 *V. cholerae*), although human-shed organisms are more infectious and require a lower inoculum [10]. Once infected, *V. cholerae* causes a spectrum of illness — from asymptomatic disease to life-threatening dehydration — depending on bacterial load, degree of background immunity and presence or absence of malnutrition [11]. The incubation period varies between hosts and inoculum size, from 1 to 5 days. Mild cases may be indistinguishable from other causes of diarrhoeal illness, while profound infection causes rapid loss of fluid and electrolytes in ‘rice water’ stool (containing large amounts of sodium, potassium and bicarbonate) at rates of 10–20 ml/kg/h [3]. Severe hypovolaemia may occur within hours of symptom onset, resulting in hypovolaemic shock, hypokalaemia, lactic acidosis (owing to bicarbonate loss), acute renal failure and hypoglycaemic coma. The mortality of untreated cholera is 50–70%, and children have a 10 times greater risk of death than adults [5].

Cholera is commonly diagnosed and treated presumptively on the basis of clinical features. It can be confirmed by isolation of *V. cholerae* from stool cultures performed on specific media (TCBS or TTGA agar), with rapid diagnostic tests also available (which tend to be highly sensitive but poorly specific, limiting their usefulness in endemic areas) [5]. The 2013 World Health Organization (WHO) Pocketbook for Hospital Care defines cholera as ‘profuse watery diarrhoea with severe dehydration’ during a cholera outbreak *or* a positive stool culture for *V. cholerae* 01 or 0139 [12].

Fluid resuscitation is the mainstay of treatment (reducing mortality to <0.5%) [1] and, while antimicrobial therapy does not have an immediate effect on disease progression (as the toxin is already bound to intestinal cells), they decrease the duration of the disease by diminishing further production of the toxin by inhibiting bacterial protein synthesis or promoting bacterial cell death [9]. Importantly in epidemics, antimicrobial therapy also diminishes pathogen excretion which reduces person-to-person transmission of infection, as well as limiting environmental contamination by cholera by diminishing the volume and duration of stools passed (by approximately 50%), shortening the period of faecal excretion of *V. cholera* [13]. Clinical recovery is therefore expedited, while the volume of rehydration fluid required (and burden on medical care) is diminished, optimising use of resources during outbreaks and decreasing the rate of infectivity [14–19].

Currently, WHO recommends antibiotics (as soon as vomiting stops, usually 4–6 h after commencing oral rehydration therapy) for children aged > 2 years with ‘severe dehydration’ (Table 1). However, the current WHO recommendations for antimicrobial therapy (Table 2) are based on evidence from 2005 [12,20]. In view of increasing antimicrobial resistance worldwide and the changing efficacy and safety profiles, this review of the international literature was undertaken to update the evidence surrounding the recommendations for antibiotic treatment in paediatric cholera infection.

**Table 1.** WHO classification of dehydration in children with cholera [2,3].

WHO classification of dehydration condition	No dehydration (fluid deficit estimated as <5% of bodyweight)	Moderate (‘SOME’) dehydration (estimated fluid deficit of 5–10% of bodyweight)	Severe dehydration (estimated fluid deficit >10% of bodyweight)
		Two or more of the below:	Two or more of the below:
	Well, alert	Restless, irritable	Lethargic or unconscious
Eyes	Normal	Sunken	Sunken
Thirst	Drinks normally, not thirsty	Thirsty, drinks easily	Drinks poorly or unable to drink
Skin ‘pinch’	Goes back quickly	Goes back slowly	Goes back very slowly
Fluid therapy	Home-based oral rehydration therapy	Reduced osmolality oral rehydration solution (ORS), rice-based ORS or amylase-resistant starch ORS	IV rehydration with isotonic fluids (Ringer solution preferred)

**Table 2.** Published WHO recommendations for antibiotic therapy for children >2 years presenting with suspected cholera.

Condition	‘Antibiotic of choice’	Alternative	In addition
WHO Pocketbook Recommendations Cholera with *severe* dehydration [12,20]	Tetracycline 12.5 mg/kg *qid* for 3 days	Erythromycin 12.5 mg/kg *qid* for 3 days	Zinc supplementation
	*or*	*or*	20 mg/kg for 10–14 days as soon as vomiting has ceased
	Doxycycline (dosage not listed)	Chloramphenicol 20 mg/kg IM *qid* for 3 days	
	*or*		
	Cotrimoxazole (dosage not listed)		
WHO 2010 PAHO Recommendations (Haiti outbreak)	Option 1	Option 2	
Children over 3 years who can swallow tablets	Erythromycin 12.5 mg/kg/6 h for 3 days	Ciprofloxacin, suspension or tablets 20 mg/kg in a single dose	
	*or*	*or*	
	Azithromycin, 20 mg/kg in a single dose not exceeding 1 g	doxycycline suspension or tablets 2–4 mg/kg PO in single dose	
Children under 3 years, or infants who cannot swallow tablets	Erythromycin, suspension,	Ciprofloxacin suspension 20 mg/kg, in a single dose	
	12.5 mg/kg/6 h for 3 days		
	*or*	*or*	
	Azithromycin suspension 20 mg/kg in a single dose	Doxycycline syrup 2–4 mg/kg PO in a single dose	

Note: IM, intramuscular; *qid*, four times daily.

## Methods

### Search terms

A systematic search of systematic reviews, meta-analyses, multi-centre studies and randomised-controlled trials for relevant papers was conducted using the MeSH Search terms ‘cholera, ‘antibiotics’ and ‘antimicrobials’. The databases EMBASE, Cochrane database of systematic reviews and PubMed were searched. Trials were limited to those in humans published in the past decade to ensure that accurate and up-to-date information on antimicrobial resistance patterns was documented. The reference lists of relevant publications were also reviewed. Inclusion and exclusion criteria are documented in Table 3.

**Table 3.** Inclusion and exclusion criteria for review of the evidence for antimicrobial treatment of cholera infection.

Inclusion criteria	Exclusion criteria
• Systematic review, randomised controlled trial or multi-centre study investigating clinical treatment options and outcomes for *V. cholerae* • Where resistance patterns were investigated, information on antimicrobial testing methodologies were clearly documented	• Published >10 years prior to search period • Not pertaining to treatment in humans • Data pertaining to carriage rates only

## Results

**Figure 1.**
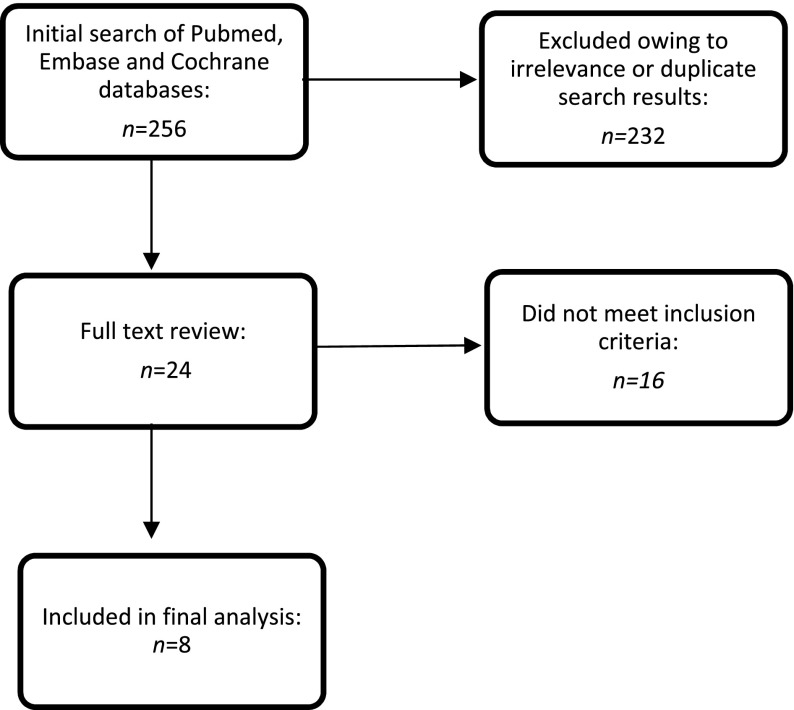
Search strategy.

The initial search produced 256 results (Figure 1), 24 of which qualified for full text review. Ultimately, eight studies met the inclusion criteria (Table 3) and were abstracted as detailed in Appendix 1. Quality assessment of the studies was performed as per the Grading of Recommendations Assessment, Development and Evaluation (GRADE) guidelines [21].

The search was initially restricted to results investigating the paediatric population, but owing to limited research in this area, it was expanded to include research in all age ranges. International clinical practice guidelines were also reviewed, including the Infectious Diseases Society of America (IDSA), World Gastroenterology Guidelines, ICDDR,B, the United States Centre for Disease Control, BMJ Clinical Evidence, the American Academy of Paediatrics, and Therapeutic Guidelines (Australia) [16,22–26].

### Characteristics of the studies included

Three studies were systematic reviews and meta-analyses, two of which were conducted across an international setting while one was conducted in sub-Saharan Africa [27]. One study was a systematic descriptive analysis (which included a systematic search) of information regarding the epidemiology of cholera outbreaks in Asia and included descriptive analyses regarding increasing antimicrobial resistance patterns [28]. There were two randomised controlled trials, one open-labelled-controlled clinical trial and one multi-centre study conducted in four sites.

Only two papers analysed the paediatric population specifically (age 2–16 years) [6,19] while the remaining systematic reviews covered all age ranges [27–29] and three clinical trials included only adults [29–31]. Most clinical trials were conducted in Asia — Bangladesh [19,29,30] and India [31]. The studies were analysed according to GRADE level of evidence criteria (see Appendix 1 for description of methodologies and relevant limitations) [21]. No studies were assessed as high-quality evidence. Three were classified as being of moderate quality [19,32,33], four as low quality [6,27,29,31] and one as very low quality [28].

### Evidence for current WHO recommendations

#### Erythromycin

Macrolides (azithromycin, clarithromycin, erythromycin and roxithromycin) have a broad spectrum of activity against Gram-positive and Gram-negative cocci (as well as Gram-negative anaerobic bacteria), attaining high intracellular concentrations beneficial for the treatment of infections caused by intracellular pathogens. As inhibitors of the cytochrome P450 (CYP3A4) enzyme system, drug interactions and adverse effects can occur (discussed below). Oral formulations of erythromycin have variable absorption and are poorly tolerated owing to adverse gastrointestinal effects, and poor adherence is exacerbated by the four times daily dosing schedule [26]. A 2014 systematic review of the evidence of two trials (involving 179 participants) showed that single-dose erythromycin was inferior to azithromycin which shortened the duration of diarrhoea by half a day compared with erythromycin [mean duration (MD) 12.05, 95% CI 22.02–2.08] [32].

Although they are outside the inclusion time-frame for this review, it is worth mentioning two studies completed in 2002 [14] and 2005 [34] which evaluated the clinical efficacy of erythromycin in childhood cholera. A double-blind randomised-controlled trial in a tertiary centre in Bangladesh of 128 children aged 1–15 years with severe dehydration treated with single-dose azithromycin (20 mg/kg) *vs* four times daily erythromycin (12.5 mg/kg) for 3 days found no significant difference in clinical success between the two groups (76% of patients receiving azithromycin *vs* 65% in the erythromycin group (95% CI 5–7, *p* = 0.24) and no significant difference in bacteriological success (71% of azithromycin group *vs* 82% of the erythromycin group, 95% CI 5–25, *p* = 0.26) [14]. Furthermore, this RCT found that patients treated with azithromycin had a significantly shorter duration of diarrhoea [median 24 h *vs* 42 h, difference 12, 95% CI {surrounding difference} 0–18 h, *p* = 0.019) and fewer episodes of vomiting (1 *vs* 4, difference = 1, 95% CI surrounding difference 0–3, *p* = 0.023) [14]. A second randomised, open-label-controlled clinical trial published in 2005 compared single-dose ciprofloxacin (20 mg/kg) with erythromycin (12.5 mg/kg four times daily for 3 days) in 180 children aged 2–15 years with *V. cholerae* infection (confirmed by stool microscopy) and found no significant difference in clinical success between children treated with erythromycin *vs* ciprofloxacin (difference 5%, 95% CI 10–21) [34]. However, children treated with ciprofloxacin had less vomiting (58 *vs* 74%, difference 16%, 95% CI 2–30%), fewer stools [15 *vs* 21%, difference 6% (95% CI 0–9%)] and less stool volume [152 *vs* 196 ml/kg, difference 43 ml/kg (95% CI 13–87)] than those treated with erythromycin, yet bacteriological failure was significantly more common in ciprofloxacin-treated patients [58 *vs* 30%, difference 28% (95% CI 13–43)] [34].

#### Ciprofloxacin

Historically, fluoroquinolones have been viewed as attractive agents for treating cholera because of their very good activity *in vitro*, high concentrations in the gut lumen, high therapeutic ratio and relatively long half-life [33]. These characteristics have led to their widespread use as single dose therapy, or as a daily dose therapy (for 3 days). However, the evidence of this review demonstrates that resistance to this class of drugs for treating cholera is increasing.

A 2014 systematic review [32] found no statistically significant difference in ciprofloxacin compared with tetracyclines in reducing the duration of diarrhoea or stool volume (three trials, 259 participants, moderate-quality evidence). A further systematic review in 2016 which assessed fluoroquinolone resistance in sub-Saharan Africa found high levels of resistance to nalidixic acid, with reduced susceptibility to ciprofloxacin observed in recent outbreaks (in the Democratic Republic of Congo, Kenya, Nigeria and Cameroon) [27]. Data from four clinical trials in adults (*n* = 275) in Bangladesh [30] also found a poor clinical response to a single, 1-g dose of ciprofloxacin (a standard treatment for adults with cholera in South-East Asia). Clinical success (defined as cessation of diarrhoea within 48 h) was achieved in only 18% of patients with nalidixic acid-resistant *V. cholerae* infection; the majority of isolates were found to be resistant [this clinical success improved to 67% in those treated with a 3-day course (difference 0.49, 95% CI −0.68 to −0.22, *p* < 0.001). This research emphasised the highly apparent increase in non-susceptibility to fluoroquinolones in the region during the study period, with a dramatically increasing MIC35 for ciprofloxacin, from 0.002 μg/ml in 1994 to 0.250 μg/ml in 2003, a 125-fold increase. Concurrently, all isolates became resistant to nalidixic acid [30].

In the 2005 randomised, open-label-controlled trial discussed above which found that single-dose ciprofloxacin had similar clinical efficacy to a 3-day course of erythromyin (60 *vs* 55%, 95% CI 10–21), bacteriological failure was more common with ciprofloxacin (58 *vs* 30%, 95% CI 13–43%) [34]. In a 2010 RCT of paediatric patients in Bangladesh, ciprofloxacin was also found to be clinically inferior to azithromycin and bacteriological success was, again, significantly less [19].

A number of authors have noted that current thresholds of antimicrobial susceptibility to ciprofloxacin are inappropriately low, with poorer clinical outcomes in isolates defined by the Clinical Laboratory Standards Institute as susceptible *in vitro* [29,30,35,36]. Owing to cross resistance with nalidixic acid (secondary to a single mutation in the *gyrA* gene coding the DNA gyrase) and the high worldwide resistance patterns to nalidixic acid [30], ciprofloxacin is not recommended for use in cholera.

#### Tetracyclines

Tetracyclines have a broad spectrum of activity that includes Gram-positive and Gram-negative bacteria. Common adverse effects (discussed below) include oesophagitis, photosensitivity and enamel dysplasia which often precludes their use in children <8 years, although the risk appears to be minimal if single short courses are used [25].

A recent systematic review assessed 39 trials in 4632 participants, and found that there was no difference in clinical outcomes between patients treated with tetracycline and those treated with doxycycline (three trials, 230 participants, very low quality evidence), or in patients treated with tetracycline compared with ciprofloxacin or norfloxacin (three trials, 259 participants, moderate quality evidence) [32]. However, in indirect comparisons with substantially more trials, tetracycline exhibited benefits over doxycycline, norfloxacin and trimethoprim–sulphamethoxazole (TMP-SMX) for the primary review outcomes (reducing stool volume, vibrio excretion and the amount of rehydration fluids required) [32]. Another systematic review [6] identified one study which compared the efficacy of tetracycline in children aged 1–5 years in Bangladesh, revealing that, compared with tetracycline, the mean total times to recovery were prolonged by 66% with placebo (*p* < 0.001), 25% with ampicillin (*p* < 0.017) and 9% with erythromycin (*p* = 0.37), yet these data were collated in 1998 and so are unlikely to represent current susceptibility patterns.

#### Cotrimoxazole

Whilst outside the time frame for this review, two trials conducted more than 20 years ago evaluated the efficacy of cotrimoxazole. Both showed no difference from other antimicrobials but were statistically inadequately powered [37,38].

### Evidence for alternative antibiotics

#### Doxycyline

As outlined above, tetracyclines exhibit clinical benefit over doxycycline [32]. Trials dated prior to this review period have found doxycycline to be inferior to alternative antibiotics (including ciprofloxacin) for treating cholera [39]. Furthermore, there is evidence that *in vitro* doxycycline susceptibilities are not a useful indicator of the *in vivo* efficacy of the drug [39], and concerns regarding their adverse effects limit its use in older children and adults.

#### Azithromycin

Four publications examined the efficacy of azithromycin in treating cholera [19,29,31,32]. A recent systematic review [32] found single-dose azithromycin to be superior to ciprofloxacin and erythromycin in shortening the duration of diarrhoea (*vs* ciprofloxaxcin, MD 32.43 h, 95% CI 62.90 to −1.95, two trials, 375 participants, moderate-quality evidence; and *vs* erythromycin MD 12.05 h, 95% CI −22.02 to −2.08, two trials, 179 participants, moderate-quality evidence). It was not compared with tetracycline.

In a 2010 clinical trial of 180 paediatric patients with cholera in Bangladesh [19], single-dose azithromycin (20 mg/kg) was compared with single-dose ciprofloxacin (20 mg/kg), and azithromycin achieved greater clinical success (defined as resolution of diarrhoea within 24 h — earlier than the usual timeline of 48 h) than ciprofloxacin (95 *vs* 70.6%, RR 1.33, 95% CI 0.65–0.86). Similar outcomes were observed for bacteriological success (defined as eradication of *V. cholerae* in the stool sample from day 3: 100% for azithromycin *vs* 96% for ciprofloxacin, RR 1.04, 95% CI 0.91–0.99, *p* = 0.06).

A 2014 RCT in 120 adult males in Kolkata compared single-dose azithromyin (1 g) with norfloxacin (400 mg) twice daily for 3 days, and found no statistically significant difference in clinical outcome (stool volume and urine output, duration of diarrhoea, total fluid requirement); the authors concluded that azithromycin is not more effective than norfloxacin [31]. However, they noted that azithromycin remained clinically superior in the paediatric age range owing to the ease of single-dosing and the availability of a syrup (norfloxacin is available only in tablets).

Further superiority of single-dose azithromycin (compared with single-dose ciprofloxacin) was also found in a 2006 double-blind RCT in 195 male adults in Bangladesh with 73% of patients achieving clinical success compared with 27% of those treated with ciprofloxacin [29]. The authors concluded that, in adults and children, single-dose azithromycin is an effective (and inexpensive) drug for the treatment of cholera caused by susceptible strains of *V. cholerae*.

Finally, in a 2002 double-blind RCT in paediatric patients slightly earlier than this search period (detailed above) in which single-dose azithromycin (20 mg/kg) was compared with 12.5 mg/kg erythromycin four times daily for 3 days, there was no significant difference in clinical or bacteriological success between the two patient groups, although patients treated with azithromycin had significantly less vomiting and a shorter duration of diarrhoea [19]. This is further evidence of the clinical efficacy of single-dose azithromycin.

There is, therefore, substantial evidence supporting the use of azithromycin for paediatric cholera. Azithromycin’s primarily trans-intestinal and biliary route of elimination results in high concentrations in the stool, and its ease of administration with a single-dose regimen and prolonged half-life (48–72 h) enhance its clinical efficacy [31,40].

### Synopsis of evidence from international guidelines

A summary of the international guidelines reviewed is presented in Table 4. While most guidelines recommend doxycycline as first-line therapy for cholera in adults, guidelines updated in the last decade cite single-dose azithromycin as the preferred first-line therapy for children [9,24,26]. Recent consensus is that, owing to diminishing susceptibility of tetracyclines, this class of antibiotic should be reserved only for epidemics in which susceptibility has been documented. While ciprofloxacin is listed as a second-line therapy in several international guidelines, in view of recent evidence reviewed above regarding increasing worldwide ciprofloxacin resistance, it is not recommended as a routine treatment of cholera in children.

**Table 4.** Summary of international guidelines on the treatment of cholera.

Guideline	Last update		Recommendations	
American Academy of Pediatrics [9]	2015		• Antimicrobial therapy should be considered for people who are moderately to severely ill • The choice of antimicrobial therapy should be made on the basis of the age of the patient as well as prevailing patterns of antimicrobial resistance	
			Doxycycline 4–6 mg/kg single dose. For use in epidemics (only) caused by susceptible isolates. *Not recommended for pregnant women and children* <*8 years*	
			Ciprofloxacin 15 mg/kg twice daily for 3 days. Note: decreased susceptibility to fluoroquinolones is associated with treatment failure. *Ciprofloxacin is not recommended for children and pregnant women*	
			Azithromycin 20 mg/kg single dose	
			Erythromycin 12.5 mg/kg four times a day for 3 days	
			Tetracycline 12.5 mg/kg four times per day for 3 days	
Therapeutic guidelines (Australia) [26]	2015		Azithromycin 20 mg/kg up to 1 g orally as a single dose	
			OR	
			Ciprofloxacin 20 mg/kg up to 1 g orally as a single dose	
British Medical Journal ‘Best Practice’ Guidelines [25]	2017		In the event of clinical failure, treatment should be guided by susceptibility testing	
			‘The correct antibiotic is chosen based on knowledge of recently isolated *V. cholerae* strains. In LMIC, antibiotic choice is likely to be limited by what is available in sufficient quantities to cope with high demand, and susceptibility testing is likely to take longer than the mean duration of illness, forcing empirical therapy’	
			Antibiotic therapy plus zinc supplementation is recommended for *ALL patient groups* (encompassing mild-to-severe dehydration)	
			• Azithromycin 20 mg/kg PO as a single dose	
			OR	
			• Tetracycline children >8 years 12.5 mg/kg PO *qid* for 3 days	
			OR	
			• Doxycycline children >8 years 6 mg/kg/day PO or IV as a single dose	
			*NB: In the UK, tetracyclines are not recommended for children aged* ≤*12 years*	
			OR	
			• Norfloxacin 7.5 mg/kg PO bd for 3 days	
			OR	
			• Trimethoprim/sulfamethoxazole 4–5 mg/kg trimethoprim PO bd for 3 days	
			OR	
			• Ciprofloxacin 20 mg/kg PO as a single dose, or for 3 days in South Asia	
			OR	
			• Erythromycin base 12.5 mg/kg PO *qid* for 3 days	
			PLUS Zinc sulphate 30 mg PO elemental zinc once daily	
Centers for Disease Control (USA) [16]	2015		The authors note that ‘although the WHO recommends the use of antibiotics for severely dehydrated patients, there is an evolving consensus that moderately dehydrated patients would also benefit, especially if they have high purging rates despite initiation of appropriate treatment’	
			• Antibiotics should be guided by local susceptibility patterns • ‘In most countries, doxycycline is recommended as first-line treatment for adults, while azithromycin is recommended as first-line for women and children’ • ‘Recently, azithromycin has been shown to be more effective than erythromycin and ciprofloxacin [4,5] and an appropriate first-line regimen for children and pregnant women.’ • Treatment with antibiotics is recommended for patients who are *severely ill* OR *moderately dehydrated, continuing to pass a large volume of stool during rehydration treatment* • Antibiotics are also recommended for all hospitalised patients	
Infectious Diseases Society of America (IDSA) [22]	2001		• Doxycycline 300 mg single dose	
			OR	
			• Tetracycline *qid* for 3 days	
			OR	
			• TMP-SMZ 160/800 mg for 3 days	
			OR	
			• Single-dose fluoroquinolone	
World gastroenterology guidelines [24]	2012		• Routine antimicrobial therapy is recommended for treatment of ‘clinically recognisable’ cholera. • The selection of an antimicrobial will depend on recent susceptibility of the pathogen in specific countries; in the absence of such information, susceptibility reports from neighbouring countries is the only other choice. • Doxycycline 2 mg/kg *(not recommended for children)* • Azithromycin 20 mg/kg as a single dose • Ciprofloxacin 15 mg/kg every 12 h for 3 days (the MIC has increased in many countries, necessitating multiple-dose therapy over 3 days) • Trimethoprim/sulfamethoxazole (TMP/SMX; 5 mg/kg TMP + 25 mg/kg SMX, 12-hourly for 3 days), and norfloxacin.	
International Centre for Diarrhoeal Disease Research (ICDDR,B) [23]	1997		Antibiotics are recommended for those with ‘clinically diagnosed cholera’, not limited by severity.	
			• Tetracycline 12.5 mg/kg *qid* for 3 days • Erythromycin 12.5 mg/kg *qid* for 3 days	

### Clinical dehydration and the indication for antibiotic treatment

Current WHO guidelines recommend antibiotics only for patients with severe dehydration. However, as outlined in Table 4, a number of international guidelines extend this to include patients with both moderate and severe dehydration [9,16,23,25] with some even indicating antibiotic therapy for patients with mild dehydration [26] or ‘clinically diagnosed cholera’, not limited by severity [23,24].

The guidelines for expanded therapy regardless of fluid status are largely based on the results of the systematic reviews discussed above which document significant clinical and bacteriological success in patients with both severe *and* moderate dehydration treated with antibiotics [6,32]. A 2014 systematic review of 39 trials in 4632 participants found that antibiotic therapy shortened the mean duration of diarrhoea by approximately 1.5 days compared with placebo or no treatment (MD 36.76 h, 95% CI −43.51 to −30.03, data from 19 trials in 1103 participants, moderate-quality evidence), reduced total stool volume by 50% (ratio of means 0.5, 95% CI 0.45–0.56, 11 trials, 1201 participants, moderate-quality evidence) and reduced mean duration of faecal excretion of vibrios by almost 3 days (MD 2.74, 95% CI −3.07 to −2.40, 740 participants, moderate-quality evidence) [32].

These clinical and public health (through diminished transmission) benefits were seen in trials recruiting only patients with severe dehydration *and* in those with less severe dehydration [32], leading the authors to conclude that, in treating cholera, similar clinical and microbiological benefits are observed in both severely and non-severely ill patients. This was substantiated by a systematic review which also concluded that antibiotics have a clinical benefit in moderately dehydrated patients with cholera, and no adverse effects of their use were identified [41].

In view of the evidence of these large systematic reviews and the international consensus in recently updated international guidelines, antibiotic therapy in cholera outbreaks should be extended, if resources allow, to all paediatric patients presenting with signs of dehydration (i.e. those requiring hospital-based oral or intravenous rehydration, defined as those with ‘some’ or ‘severe’ dehydration as per the WHO 2005 guidelines; Table 2) [13].

### Evidence regarding the duration of antibiotic therapy

The duration of antimicrobial therapy depends on the choice of antibiotic (Table 4 and 5). Erythromycin and tetracycline require 3-day courses for bacteriological success, and, while doxycycline may be a useful single dose therapy in susceptible epidemics, resistance is increasing and its use should be limited to older children [32]. As outlined above, the most promising evidence in the paediatric age range for single-dose therapy is for azithromycin because increasing minimal inhibitory concentrations (MICs) for ciprofloxacin now mean that it is not effective as a single-dose [29,32]. Single-dose therapy has significant advantages: compliance is assured (and the development of resistance is, therefore, diminished), treatment is more affordable and logistics are improved, an important point when considering treatment strategies in rapidly spreading cholera epidemics [23,33].

**Table 5.** Recommended duration and dosage of evidence-based antibiotics to treat cholera in children.

Antibiotic	Dosage	Frequency	Duration of therapy	Notes
Ciprofloxacin	15 mg/kg	Twice daily	3 days	Increasing frequency and duration of therapy recommended due to increasing MICs [6]
Azithromycin	20 mg/kg	Single dose	Single dose	Recommended first-line therapy
Erythromycin	12.5 mg/kg	Four times daily	3 days	Recommended second-line therapy
Tetracycline	12.5 mg/kg	Four times daily	3 days	For children >12 years, increasing global resistance
Doxycycline	6 mg/kg	Single dose	Single Dose	For children >12 years, increasing global resistance

**Table 6.** Common adverse reactions to antibiotics currently indicated to treat cholera in children.

Antibiotic	Life-threatening	Mild adverse effects which may result in discontinuation of treatment	Other	Relevant interactions
Tetracyclines, including doxycycline	Hypersensitivity reactions; anaphylaxis	Photosensitivity; diarrhoea; nausea; oesophageal irritation	Benign intracranial hypertension Deposition in developing bone and teeth by binding to calcium, which can cause dental staining and hypoplasia in children <12 years	• Zinc, antacids, calcium, magnesium and iron all decrease the absorption of tetracyclines; *of importance when zinc*-*containing products are used as adjunctive therapies for treating diarrhoea in children* • Contra-indicated in pregnancy and breast-feeding
*Fluoroquinolones* Ciprofloxacin Norfloxacin Ofloxacin	Hypersensitivity reactions; Prolonged QT syndrome	Dyspepsia, headache, diarrhoea, vomiting, hypotension	Tendonitis and tendon rupture; Peripheral neuropathy	• All fluoroquinolones should be used with caution in patients receiving drugs known to prolong the QT interval • The toxicity of fluoroquinolones is increased by the concurrent use of systemic steroidal medications • Fluoroquinolones’ effects are reduced by the co-administration of iron- and zinc- containing products, of importance when zinc-containing products are used as adjunctive therapies for treating diarrhoea in children • Fluoroquinolones cause additive toxicity with non-steroidal anti-inflammatory drugs (ibuprofen, meloxicam, naproxen)
*MACROLIDES*: Erythromycin Azithromycin	Hypersensitivity Reactions; Prolonged QT syndrome	Dyspepsia, flatulence, headache, disturbance in taste, anorexia, diarrhoea, vomiting# *Gastrointestinal side effects are less significant with azithromycin than erythromycin*	Malaise, Paresthesia Risk of pyloric stenosis in neonates	• All macrolides are advised to be avoided concomitantly with other drugs which prolong the QT interval • Plasma concentrations of azithromycin are increased by ritonavir • Azithromycin in combination with rifabutin results in increased side effects of ritabutin, including neutropenia

Note: LMIC, low- and middle-income countries.

### Reviews of harms and toxicity — summary of the evidence on safety

#### Common adverse effects

Common adverse effects of the currently recommended therapies for treating cholera and those which may be relevant when updating guidelines are detailed in Table 6.

#### Prolongation of the QT interval

Published case reports suggest that fluoroquinolones and macrolides are associated with prolongation of the QT interval [42,43]. Independently, mild delays in ventricular repolarisation are clinically unnoticeable, though these antimicrobials may serve to amplify the risk of ‘torsades de pointes (TdP)’, a potentially fatal polymorphic ventricular tachyarrhythmia which may present as sudden death (owing to ventricular tachycardia), syncope, palpitations, seizures, or asymptomatically *if the duration is short and terminates spontaneously* [45]. Of note, the current literature identifies this risk as requiring the presence of other risk factors, as highlighted in Table 7.

**Table 6.** Risk factors for the development of torsades de pointes.

Risk factor	Examples
Genetic risk factors	Channelopathies
	CYP3A4 poor metaboliser

Underlying cardiac disease	Bradycardia
	Congestive cardiac failure
	Myocardial ischaemia
	Atrial fibrillation

Electrolyte derangements	Hypokalaemia
	Hypomagnesaemia
	Hypocalcaemia

Organ impairment, altering medication toxicity	Renal insufficiency
	Severe hepatic disease

Use of medication to increase QT liability	Concurrent CYP medications administered

The predominant risk factor for the development of TdP is co-administration of other medications which are substrates and/or inhibitors of cytochrome P450 (CYP) enzymes, and the associated ‘metabolic liability’ resulting from co-administration of medications synergistically interacting with this enzyme. This risk is enhanced by individual allelic variations in CYP3A4, the most important enzyme in human drug metabolism. CYP3A4 is responsible for the biotransformation of approximately 60% of all oxidised drugs [44] and allelic variations can result in patients being poor metabolisers of CYP3A4-inducing medications [45], resulting in reduced clearance of drug substrates and increasing exposure to toxicity effects. Overall, the individual risk of cardiac arrhythmias secondary to these antimicrobials is minimal; yet, if combined with a genetic propensity to poor metabolism of CYP3A4-inducing medications and co-administration with other CYP potentiators, the risk may be magnified, although the clinical impact of this is unknown.

#### Prolonged QT syndrome and azithromycin

Azithromycin has been identified as being distinguishable from macrolides as a group in terms of its cardiac toxicity, as it minimally inhibits CYP3A4, resulting in a lack of appreciable interaction with other CYP3A4 substrates. It is therefore classified as one of the safer macrolide antibiotics from a cardiac perspective [45,47]. In recent years, however, increasing attention has been paid to azithromycin’s risks following a documented increased risk of cardiac death in a cohort of 347,795 patients aged 30–74 years taking azithromycin. The study found that patients taking 5 days of azithromycin compared with taking no antibiotics had a statistically significant increased risk of cardiac death [hazard ratio (HR) 2.88, 95% CI 1.25–2.75, *p* < 0.0001] as well as death from any cause (HR 1.85, 95% CI 1.25–2.75, *p* = 0.002). However, the risk was found to be most pronounced in patients with a high baseline risk of cardiovascular disease, and there was evidence of confounding by factors associated with both azithromycin use and risk of cardiovascular disease — namely a history of smoking, high body mass index, poor diet, and low physical activity [46]. At present, published case reports of increased risk of sudden cardiac deaths in patients taking azithromycin are limited to adults, and whether these findings apply to the paediatric population cannot be concluded [47].

A considerable risk in severe cholera is that of hypovolaemia-induced hypokalaemia owing to potassium loss in the stool, which in itself is a risk factor for arrhythmias (specifically, a prolonged PR interval and flattened T-waves) [25]. As such, adequate fluid replacement with potassium-containing oral and intravenous solutions should remain of paramount importance in treating patients with cholera to minimise the possibility of this risk factor contributing to the risk of TdP.

#### Prolonged QT syndrome and fluoroquinolones

As with macrolides, there is interclass variability in the QT prolongation effect of fluoroquinolones. Ciprofloxacin’s inhibition of CYP1A2 has been described as ‘relatively inconsequential’ [45], and the US Food and Drug Administration (FDA)’s Adverse Event Reporting System (AERS) supports the notion of multifactorial causes of fluoroquinolone-associated TdP, usually occurring in the context of co-administration with another QT-prolonging drug, underlying cardiac disease, renal impairment and electrolyte anomaly. However, combined with the increasing resistance of cholera to ciprofloxacin and the longer course that is required to overcome increasing MICs, ciprofloxacin should not be recommended as a first-line therapy for treating paediatric cholera.

### Gastrointestinal side effects of macrolide administration

Previous clinical trials have documented significantly less vomiting in patients treated with azithromycin compared with erythromycin (1 *vs* 4, difference one episode, 95% CI 0–3 episodes, *p* = 0.023) [19]. While vomiting is also a manifestation of cholera, the difference in the number of episodes of vomiting suggests that prolonged vomiting in patients treated with erythromycin may be attributed to an adverse effect rather than to the disease process itself. Azithromycin is therefore considered clinically superior to erythromycin because of its short-course requirement and subsequently diminished risk of gastro-intestinal side effects.

### Antibiotic resistance and chemoprophylaxis regimens

Increasing the administration of antibiotics to children with less severe dehydration needs to be weighed against the effect it may have on antibiotic resistance in cholera. Alongside the clinical efficacy data discussed above, laboratory-based studies in Asia have found high levels of multi-drug resistance in strains of *V. cholerae* 01 in the past decade. A laboratory analysis of 302 strains associated with endemic cholera in Thailand found that 71% were resistant to erythromycin, 54% TMP-SMX, 23% to tetracycline and 31% to ampicillin, with 23% of the strains exhibiting multi-drug resistance [48]. A 2012 study of 100 isolates in Vietnam (collected between 2007 and 2010) found all isolates were completely resistant to TMP-SMX and nalidixic acid, 29% were resistant to tetracycline and 85% exhibited multi-drug resistance (to nalidixic acid, TMP-SMX and tetracyclines), yet there was 95% susceptibility to azithromycin [49]. Similarly, high levels of erythromycin and tetracycline resistance have been documented in laboratories in Dhaka [50], while a laboratory analysis of 77 rectal swabs from patients presenting during cholera epidemics in Mozambique found high incidences of resistance to chloramphenicol (58%), TMP-SMX (97%) and tetracycline (97%) (yet quinolone resistance remained low at 4.2%) [51].

These increasing resistance patterns must be taken into account when considering the appropriate first-line therapy for paediatric cholera and other interventions, such as the administration of chemoprophylaxis for contacts of patients with cholera. A systematic review and meta-analysis in 2011 found that chemoprophylaxis reduced infectivity rates (RR 0.39, 95% CI 0.29–0.51) and hospitalisation of contacts (RR 0.54, 95% CI 0.4–0.74) [52], yet mass prophylaxis may lead to rising resistance rates in isolates, causing subsequently resistant clinical cases [9,10]. Although families of patients with cholera are at high risk of contracting cholera themselves, they should receive targeted education about safe water and sanitation, plus appropriate administration of oral rehydration solution, rather than prophylactic antibiotic therapy.

## Discussion

Cholera is an important cause of diarrhoeal illness, and the burden it imposes has increased over the past decade [6]. It is responsible for 3 million cases and 100,000 deaths worldwide each year [10], and 1.4 billion people live in places where cholera is endemic [9]. Prevention of infection through adequate sanitation and access to clean water is paramount, and the cornerstone of treatment remains access to aggressive fluid rehydration which reduces mortality to <0.5% [16].

Antimicrobial therapy decreases further production of the cholera toxin, and the current international literature supports antibiotic treatment of children with dehydration who require hospital admission during epidemics, when resources allow [32]. The evidence demonstrates that antibiotic therapy reduces the volume of stool passed which diminishes the volume of rehydration required, minimises the burden on medical care in resource-constrained settings and reduces the transmission of infection.

The 2005 WHO guidelines listed tetracycline (12.5 mg/kg *qid* for 3 days) as the treatment of choice for children >2 years with severe dehydration, with an expanded list of antimicrobial choices published in the 2013 Pocketbook of Hospital Care for Sick Children (including doxycycline, TMP-SMX, erythromycin and chloramphenicol alongside zinc supplementation once vomiting has stopped) [12]. However, this review has found increasing evidence of resistance to tetracyclines and ciprofloxacin for cholera infection in adults and children.

Alongside their patterns of increasing resistance, tetracycline antimicrobials are contraindicated in young children in higher income settings owing to their adverse effects.

The macrolide azithromycin has been shown to be clinically superior to tetracyclines in treating cholera infection in children, and the benefits of instituting this as first-line therapy in treating cholera outweigh the limited evidence to suggest macrolides are associated with cardiac arrhythmias by prolonging the QT interval. Moreover, in its class of antibiotics, azithromycin has been distinguished as one of the safest macrolides in terms of its cardiac side-effects. While single-dose erythromycin is inferior to azithromycin, when administered four times daily, it has been shown to exhibit similar clinical efficacy and bacteriological success in treating children with cholera [46,47], although the regular and prolonged (3-day) course required makes adherence challenging. However, erythromycin is clinically superior to ciprofloxacin as an alternative therapy for cholera, and because of its lower cost and improved bacteriological clearance rates [22,47] it is an appropriate second-line therapy for cholera in children, although the increasing resistance needs to be closely monitored.

Microscopy and susceptibility testing conducted in laboratories with external quality assurance should continue to be of paramount importance prior to commencing therapy, and if this is not locally available susceptibility testing from neighbouring regions should be used. Future research should continue to monitor the resistance profiles of antimicrobials used to treat cholera infection to diminish the spread of further antimicrobial resistance in *V. cholerae* infection, and monitor adverse effects of antimicrobials used to treat cholera infection in the paediatric population.

## Disclosure statement

No potential conflict of interest was reported by the authors.

## Funding

This work was supported by the World Health Organisation; The Nuffield Department of Medicine (The University of Oxford); General Sir John Monash Foundation; The Wellcome Trust [grant number MR/M007367/1]; and the Bill and Melinda Gates Foundation [grant number OPP1131320].

## Notes on contributors

***Phoebe C. M. Williams***, MBBS(Hons.), received her medical degree from the University of Sydney and a master’s in Global Health Science from the University of Oxford. She is a paediatric registrar and dual trainee in Infectious Diseases at Sydney Children’s Hospital, Australia. She is a DPhil candidate through the University of Oxford, with her research focusing on antimicrobial resistance in paediatric patients.

***James A. Berkley*** FRCPCH, MD is a professor of Paediatric Infectious Diseases at the University of Oxford based at the KEMRI–Wellcome Trust Research Programme in Kilifi, Kenya. He is the principal investigator of the CHAIN network with a research focus on serious infection and survival in highly vulnerable groups of infants and children.

## Appendix 1

**Table:**

Authors	Year	Title	Methods, setting and study limitations	Results	Conclusion	Level of evidence
Leibovici-Weissman Y, Neuberger A, Bitterman R, et al. [1]	2014	Antimicrobial drugs for treating cholera (review)	• Systematic review and meta-analysis • All age ranges • Search included Cochrane, CENTRAL, PubMed, EMBASE, African Index Medicus, LILACS, Science Citation Index, metaRegister of Controlled Trials, WHO International Clinical Trials Registry Platform, conference proceedings and reference lists; to March 2014 • Selection criteria: Randomised and quasi-randomised controlled clinical trials in *adults and children* with cholera that compared: ◦ Any antimicrobial treatment with placebo or no treatment; ◦ Different antimicrobials head-to-head; or ◦ Different dosing schedules or different durations of treatment with the same antimicrobial • Diarrhoea duration and stool volume were defined as primary outcomes • The mean difference (MD) or ratio of means (ROM) were calculated for continuous outcomes, with 95% CI and pooled data using a random-effects meta-analysis • The quality of evidence was assessed using the GRADE approach	• 39 trials were included in this review with 4623 participants • Overall, antimicrobial therapy *shortened the mean duration of diarrhoea by approximately 1.5 days* compared with placebo or no treatment (MD 36.77 h, 95% CI −43.51 to −30.03, 19 trials, 1013 participants, moderate-quality evidence). • Antimicrobial therapy also *reduced the total stool volume* by 50% (ROM 0.5, 95% CI 0.45 to 0.56, 18 trials, 1042 participants, moderate-quality evidence) and *reduced the amount of rehydration fluids required* by 40% (ROM 0.60, 95% CI 0.53–0.68, 11 trials, 1201 participants, moderate-quality evidence) • The *mean duration of faecal excretion* of vibrios was *reduced by almost 3 days* (MD 2.74 days, 95% CI −3.07 to - 2.40, 12 trials, 740 participants, moderate-quality evidence) • There was *substantial heterogeneity* in the size of these benefits, probably owing to *differences in the antibiotic used, the trial methods (particularly effective randomisation) and the timing of outcome assessment* • The benefits of antibiotics were seen both in trials recruiting only patients with severe dehydration and in those recruiting patients with mixed levels of dehydration • *Comparisons of antimicrobials:* In head-to-head comparisons, *no differences* were detected in diarrhoea duration or stool volume for *tetracycline compared with doxycycline* (three trials, 230 participants, *very low-quality evidence*); or *tetracycline compared with ciprofloxacin or norfloxacin* (3 trials, 259 participants, *moderate-quality evidence*) • In *indirect comparisons* with substantially more trials, *tetracycline appeared to have greater benefits than doxycycline, norfloxacin and trimethoprim-sulfamethoxazole* for the primary review outcomes • *Single-dose azithromycin* shortened the duration of diarrhoea by over a day *compared with ciprofloxacin* (MD −32.43, 95% CI −62.90 to −1.95, two trials, 375 participants, moderate-quality evidence) and by *half a day compared with erythromycin* (MD −12.05, 95% CI - 22.02 to −2.08, two trials, 179 participants, moderate-quality evidence). It was not compared with tetracycline	• In treating cholera, antimicrobials result in substantial improvements in clinical and microbiological outcomes, with similar effects observed in severely and non-severely ill patients • *Azithromycin and tetracycline may have some advantages over other antibiotics*	B
Das J, Salam R, Bhutta Z [2]	2013	Antibiotics for the treatment of cholera, *Shigella* and *Cryptosporidium* in children	• Systematic review which included 2 studies from Bangladesh only • Children <16 years • Search covered PubMed, Cochrane, Embase and WHO Regional databases for literature published up to February 2012 to identify studies describing the effectiveness of antibiotics for the treatment of cholera in children ≤5 years; following CHERG systematic review guidelines • Additional studies were identified by hand-searching references from included studies • Search terms for cholera included combinations of ‘cholera’, ‘diarrhea’, ‘antibiotics’ • No language or date restrictions were applied • Inclusion criteria: Studies were included if they reported the effect of antibiotics on morbidity and mortality associated with diarrhoea owing to cholera in children, as observed by clinical and bacteriological failure and mortality • Only studies with a placebo group or no antibiotic control group were included • Only studies with a confirmed diagnosis of the infection and on immunocompetent patients were included	• 374 titles were identified, 21 of which were reviewed and two included in the final dataset (the only two studies with a suitable control or placebo group assessing children up to 16 years of age). Both studies were RCTs conducted in *Bangladesh (both hospital-based)* • One trial compared *erythromycin, ampicillin and tetracycline*, and the other compared *erythromycin and trimethoprim/sulfamethoxazole (TMP/SMX)* against a placebo group • Antibiotics reduce clinical signs of 63% (CI 29–81%) of cholera cases, with a RR of 0.37 (0.19–0.71) • Antibiotics successfully cleared cholera pathogens in 75% (47–88%) of cases; RR 0.25 (0.12–0.53)	• ‘Antibiotics for cholera reduce the clinical and bacteriological failure rates; however the evidence for reducing morbidity in children in insufficient to recommend antibiotic use in all cases’ • However: *included studies were >10 years old and may not represent current AMR patterns* • ‘Although the evidence is weak as there are a few studies evaluated and more research is needed, we propose that antibiotics have a potential in moderate and severe Cholera’ • No adverse events were identified by any study	C
Chattaway M, Aboderin A, Fashae K, et al. [3]	2016	Fluoroquinolone-resistant enteric bacteria in sub-Saharan Africa: clones, implications and research needs	• Systematic review • Sub-Saharan Africa • All ages • Conducted according to PRISMA guidelines • Databases searched: PubMed, AJOL databases until October 2015. 43 studies met inclusion criteria, all from 17 African countries • The search retrieved articles focused on cholera as well as *E. coli*, other enterobacteriaceae and Campylobacter • 6 papers were included which assessed resistance of cholera to fluoroquinolones • Fluoroquinolone resistance was defined as those with MIC above the CLSI breakpoints	• Despite toxigenic cholera strains becoming increasingly problematic across Africa in the past two decades, the authors note that fluoroquinolone resistance has been studied only recently • A study of the cholera outbreak in Nigeria/Cameroon in 2009 found resistance to nalidixic acid, and MICs to ciprofloxacin were 0.25–0.5 µg/mL (placing them in the susceptible category) • High levels of resistance to nalidixic acid or reduced susceptibility to ciprofloxacin in likely similar *V. cholerae* 01 clones causing epidemics in DRC and Kenya were also noted (Mercy et al., 2014; Miwanda et al., 2015; Table 1), but a lack of standard methodology for clonal analysis prevents an understanding of clonal spread across Africa	• Methods to identify fluroquinolone-resistant bacterial clones across Africa vary, making between-study and cross-country comparisons difficult • For toxigenic *V. cholerae*, serotyping and biotyping are only occasionally performed outside clinical reference laboratories • Resistance to nalidixic acid and susceptibility or reduced susceptibility to ciprofloxacin was reported in outbreaks in Africa in the past decade • Although ciprofloxacin has only reduced susceptibility in these strains and continues to be used for cholera management, if additional mutations occur in these circulating clones, resistance to ciprofloxacin may develop	C
Mahapatra T, Mahapatra S, Babu G, et al. [4]	2014	Cholera outbreaks in South and South-East Asia: descriptive analysis, 2003–2012.	• A descriptive analysis conducted following a systematic search • South and SE Asia • All ages • Review of information regarding the epidemiology of cholera outbreaks in South and Southeast Asia 2003–2012 • 58 articles analysed, 8 reports and WHO databases • PubMed and Google Scholar were searched using MeSH terms cholera, disease, outbreaks • Included studies published 2003–2012	• 66 articles met the inclusion criteria • 113 cholera outbreaks were studied, 69% in South-east Asia (52% of which occurred in India), the remainder in Asia • Several genotypes and phenotypes were identified, including *V. cholerae* 01 E Tor (Ogawa and Inaba) and *V. cholerae* 0139 • 3 studies (in Vietnam, Dhaka and Bangladesh) identified issues of multi-drug resistance, and the number of isolates with resistance was described as increasing [4–6]. These papers are discussed in the main paper (Antimicrobial resistance)	• Qualitative description of multi-drug resistance only; no quantitative data apart from individual laboratory analysis of antibiotic non-susceptibility (not clinically correlated)	D
Khan W, Saha D, Ahmed S, et al. [5]	2015	Efficacy of ciprofloxacin for treatment of cholera associated with diminished susceptibility of ciprofloxacin to *Vibrio cholerae* 01	• Adult male patients • Bangladesh • Assessed data from 4 clinical trials of antimicrobial agents in the treatment of cholera conducted between 1992 and 2005 were examined, comparing single or multiple-dose ciprofloxacin • Clinical cure was defined as cessation of watery stools within 48 h of initiation of antimicrobial therapy • Bacterial cure was defined as inability to isolate *V. cholerae* after 48 h of administration of study medication (single or multiple-dose ciprofloxacin) • *V. cholerae* were isolated and identified by standard microbiological techniques using disc-diffusion method using nalidixic acid and ciprofloxacin disks according to NCCLS/CLSI methods • Clinical response was compared with *V. cholerae* 01 susceptibility	• All 275 strains of *V. cholerae* 01 collected were susceptible to ciprofloxacin by MIC and disc diffusion testing using standard threshold criteria; *however, the MIC* ^*50*^ *and MIC* ^*90*^ *for ciprofloxacin increased significantly during this period*, from 0.002 μg/mL in 1994 to 0.250 μg/mL in 2003 (a 125-fold increase) and *MIC* ^*90*^ during the same period from 0.010 μg/mL to 0.250 μg/mL (a 25-fold increase) • During this period, all isolates also became resistant to nalidixic acid • Isolates resistant by disc-diffusion to nalidixic acid (*n* = 167) had a median ciprofloxacin MIC of 0.190 μg/mL (10th–90th centiles 0.022–0.380) compared with 0.002 μg/mL for nalidixic acid-susceptible (*n* = 38) isolates (10th–90th centiles 0.002–0.012) • Ciprofloxacin treatment was dramatically more effective in patients infected with nalidixic acid-susceptible strains of *V. cholerae*. The rate of clinical success was 95% compared with 27% in those infected with nalidixic acid-resistant isolates (*p* < 0.001) and the rate of bacteriological success was 97 *vs* 17% (*p*<0.001) • The group with infection resistant to nalidixic acid also fared worse on all secondary measures of disease outcome — diarrhoea duration, volume of stool and volume of fluids required • Single-dose ciprofloxacin was significantly inferior in treating patients with nalidixic acid-resistant *V. cholerae* infection: clinical success was achieved in only 18% of patients with nalidixic acid-resistant *V. cholerae* 01 infections treated with a single dose compared with 67%of those who received 3-day therapy	• *V. cholerae* 01 is becoming less susceptible to ciprofloxacin in Bangladesh (45-fold increase over 19 years covered in this study) • *Current thresholds* for determining antimicrobial susceptibility of *V. cholerae* to ciprofloxacin *in vitro* are *not predictive of clinical response to therapy* • Determining susceptibility to nalidixic acid using the disc diffusion method is a good screening tool for identifying *V. cholerae* 01 strains with diminished susceptibility to ciprofloxacin • Decreased resistance to fluoroquinolones is almost invariably associated with frank resistance to nalidixic acid, and usually results from a single mutation to the *gyrA* gene coding the enzyme-DNA gyrase, the target for the quinolones, although additional mutations (either in *gyrA* or other genes encoding fluoroquinolone targets) is required for frank resistance to fluoroquinolones. • The sub-optimal clinical response in patients infected with strains of *V. cholerae* 01 resistant to nalidixic acid and with diminished susceptibility to ciprofloxacin is *worse with short-course therapy*	B
Bhattaharya M, Kanungo S, Ramamurthy T, et al. [6]	2014	Comparison between single-dose azithromycin and six dose, 3 day norfloxacin for treatment of cholera in adults.Int J Biomed Sci. 2014;10:248–251. Publisjed 15 December 2014	• RCT • 120 male adults • India • Patients with acute watery diarrhoea and mod-severe dehydration compared the efficacy of 1 g azithromycin (single dose) *vs* 400 mg norfloxacin bd for 3 days in Kolkata, India (Oct 2010–Feb 2012) • Data were analysed for 64 patients who were stool culture-positive for *Vibrio cholera* (large loss to follow-up)	• There were *statistically insignificant* differences between total stool output, total duration of diarrhoea after starting treatment, total fluid requirement and total urine output between the 2 treatment groups	• Azithromycin is as effective as norfloxacin (and may be clinically superior to norfloxacin owing to its single-dosing regimen)	C
Kaushik J, Gupta P, Faridi M, et al. [7]	2010	Single-dose azithromycin *vs* ciprofloxacin for cholera in children: a randomised controlled trial	• Open-labelled clinical controlled randomised trial • Children aged 2–12 years • Bangladesh • 180 *V. cholerae* positive patients with watery diarrhoea for <24/24 *and severe dehydration* • Single dose azithromycin 20 mg/kg (*n* = 91) was compared with single-dose ciprofloxacin (20 mg/kg) (*n* = 89) • Clinical success defined as resolution of diarrhoea within 24 h • Bacteriological success defined as resolution of *V. cholerae* in the stool sample from day 3 onwards • *Exclusion criteria*: Children with severe malnutrition, coexisting systemic illness, blood in stool or having received treatment with an antibiotic within 24 h were excluded	• Frequency of stool and vomiting was significantly lower in children receiving azithromycin *vs* ciprofloxacin in the first 72 h • The rate of decline in frequency of stool and vomiting was comparable between treatment groups • Clinical success: ciprofloxacin 70.6%, azithromycin 95%, RR 1.33 (0.65–0.86, *p*<0.001) • Bacteriological success: ciprofloxacin 96%, azithromycin 100%, RR 1.04 (0.91–0.99, *p* = 0.06)	• Single-dose azithromycin is superior to single-dose ciprofloxacin for cholera in children • Clinical success was significantly greater in patients treated with azithromycin than in those treated with ciprofloxacin, although the rate of bacteriological success was comparable between the two groups • Those who received azithromycin had a shorter duration of diarrhoea (*p*<0.001), shorter excretion of *V. cholerae* (*p*<0.001) and lower requirement for IVF (*p*<0.001)	B
Saha D, Karim M, Khan W, et al. [8]	2006	Single-dose azithromycin for the treatment of cholera in adults	• Double-blind RCT comparing equivalence of azithromycin and ciprofloxacin (1 g) in 195 men with severe cholera caused by *V. cholerae* 01 or 0139 in Bangladesh • 195 male adults • Bangladesh	• Clinical success in 73% of patients receiving azithromycin and 27% of patients receiving ciprofloxacin • Patients treated with azithromycin had a shorter duration of diarrhoea than patients treated with cipro (30 *vs* 78 h) and fewer stools (36 *vs* 52) • The median MIC of cipro for the 177 isolates of *V. cholerae* 01 was 0.25 μg/ml which was 11–83 times higher than in previous studies at this site	• Single-dose *azithromycin was effective* in treating severe cholera in adults • Single-dose *ciprofloxacin is clinically and bacteriologically ineffective* in cholera caused by strains of *V. cholerae* 01 which have diminished *in vitro* susceptibility to ciprofloxacin • The *current thresholds of antimicrobial susceptibility to ciprofloxacin* may be inappropriate for *V. cholerae* 01 • The lack of efficacy of ciprofloxacin may result from its diminished activity against *V. cholerae* 01 strains • Single-dose azithromycin is therefore established as an effective drug for the treatment of cholera caused by susceptible strains of *V. cholearae* in adults and children	C
